# RecA Proteins from *Deinococcus geothermalis *and *Deinococcus murrayi *- Cloning, Purification and Biochemical Characterisation

**DOI:** 10.1186/1471-2199-12-17

**Published:** 2011-04-22

**Authors:** Marta Wanarska, Beata Krawczyk, Piotr Hildebrandt, Józef Kur

**Affiliations:** 1 Department of Microbiology, Chemical Faculty, Gdańsk University of Technology, Narutowicza 11/12, 80-233 Gdańsk, Poland

## Abstract

**Background:**

*Escherichia coli *RecA plays a crucial role in recombinational processes, the induction of SOS responses and mutagenic lesion bypasses. It has also been demonstrated that RecA protein is indispensable when it comes to the reassembly of shattered chromosomes in γ-irradiated *Deinococcus radiodurans*, one of the most radiation-resistant organisms known. Moreover, some functional differences between *E. coli *and *D. radiodurans *RecA proteins have also been shown.

**Results:**

In this study, *recA *genes from *Deinococcus geothermalis *and *Deinococcus murrayi*, bacteria that are slightly thermophilic and extremely γ-radiation resistant, were isolated, cloned and expressed in *E. coli*. After production and purification, the biochemical properties of *Dge*RecA and *Dmu*RecA proteins were determined. Both proteins continued to exist in the solutions as heterogenous populations of oligomeric forms. The DNA binding by *Dge*RecA and *Dmu*RecA proteins is stimulated by Mg^2+ ^ions. Furthermore, both proteins bind more readily to ssDNA when ssDNA and dsDNA are in the same reaction mixture. Both proteins are slightly thermostable and were completely inactivated in 10 s at 80°C. Both proteins hydrolyze ATP and dATP in the presence of ssDNA or complementary ssDNA and dsDNA, but not in the absence of DNA or in the presence of dsDNA only, and dATP was hydrolyzed more rapidly than ATP. They were also able to promote DNA strand exchange reactions by a pathway common for other RecA proteins. However, we did not obtain DNA strand exchange products when reactions were performed on an inverse pathway, characteristic for RecA of *D. radiodurans*.

**Conclusions:**

The characterization of *Dge*RecA and *Dmu*RecA proteins made in this study indicates that the unique properties of *D. radiodurans *RecA are probably not common among RecA proteins from *Deinococcus *sp.

## Background

*Deinococcus geothermalis *DSM 11302 and *Deinococcus murrayi *DSM 11303 are gram positive, nonmotile, spherical bacteria living in aerobic conditions. Cells that divide as tetrads are very common in both species. *D. geothermalis *and *D. murrayi *form orange-pigmented colonies, are slightly thermophilic with an optimum growth temperature of between 45-50°C, but differ in optimum pH for growth. *D. geothermalis *DSM 11302 is slightly acidophilic and grows optimally at pH 6.5, while *D. murrayi *DSM 11303 is slightly alcaliphilic with an optimum pH for growth of 8.0, although both species were isolated from the hot springs which had alkaline pH values ranging from 8.6 to 8.9. *D. geothermalis *and *D. murrayi *were isolated from hot springs at São Pedro do Sul and Alcafache in central Portugal, respectively. The isolation of the acidophilic *D. geothermalis *strain from an alkaline site suggests that it can colonize the microenvironments of alkaline hot springs, such as biofilms, where the pH is lowered by other microorganisms [1]. *D. geothermalis *is also able to grow on metallic surfaces of printing paper machines and it is known as an efficient primary biofilm, formerly functioning as an adhesion platform for secondary biofilm bacteria [2-4]. *D. geothermalis *and *D. murrayi *display an increased gamma radiation resistance, as would normally be found in the genus *Deinococcus *[1]. The ability of these species to withstand high doses of ionizing radiation might result from an efficient RecA-dependent DSB repair system, similar to that recently described in *Deinococcus radiodurans *[5-7].

RecA protein is a crucial DNA dependent ATPase involved in DNA repair and homologous recombination. RecA proteins are found in most microorganisms within the Bacteria domain, but some insects' and clams' endocellular bacterial symbionts such as *Buchnera aphidicola *APS, *B. aphidicola *Sg, *Blochmannia floridanus*, *B. pennsylvanicus*, *Wigglesworthia glossinidia*, *Vesiomyosocius okutanii *and *Ruthia magnifica *lack the *recA *gene [8-13]. Its analogues such as RadA [14] or Rad51 [15,16] are common in Archaea and Eucarya domains organisms. The product of the *uvsX *gene of the bacteriophage T4 also displays many RecA-like properties [17].

RecA protein of *E. coli*, the best characterized RecA, is a multifunctional protein involved in homologous recombination [18], recombinational DNA repair [19] and SOS response to DNA damage and arrest of DNA replication [20]. RecA filaments on ssDNA acts as a coprotease which facilitates the autoproteolysis of LexA protein. This results in the derepression of genes in the SOS regulon [21,22]. RecA coprotease activity also facilitates the autocatalytic cleavage of the UmuD protein to the activated UmuD', a component of DNA polymerase V (UmuD'_2_C) [23-27]. Moreover RecA nucleoprotein filament transfers RecA-ATP complex to polymerase V to form an active mutasome UmuD'_2_C-RecA-ATP which catalyzes translesion DNA synthesis [28].

*In vitro*, in the presence of Mg^2+ ^ions and ATP, dATP or nonhydrolyzable ATP analogue ATP-γ-S, RecA assembles around single-stranded DNA into a catalycally active helical filaments [29-32]. No ATP or dATP hydrolysis is needed for nucleoprotein filament formation, although these nucleotides are hydrolyzed by RecA in the presence of ssDNA [33], during the disassembly of filaments [34]. The active RecA filament is able to search out a homology between bound, single-stranded DNA and double-stranded molecules, and catalyzes the homologous pairing of DNA stands. These reactions also do not require cofactor hydrolysis and can occur in the presence of ATP-γ-S [35,36]. The RecA protein of *E. coli *promotes both three-strand exchange reaction between homologous ssDNA and dsDNA molecules, and four-strand exchange between a duplex DNA with a single-stranded tail and a full dsDNA, where the strand exchange reaction is initiated in the single-stranded region [37,38]. Although homologous pairing and DNA strand exchange can occur in the three-strand exchange reaction without ATP hydrolysis [36], ATP hydrolysis renders RecA protein-mediated DNA strand exchange unidirectional (5' to 3' with respect to the single-stranded DNA). In the presence of ATP-γ-S DNA strand exchange is bidirectional and limited in extent [39]. Moreover, ATP hydrolysis allows a heterologous sequence bypass in one of the DNA substrates [40,41], and is indispensable in the four-strand exchange reaction [41,42]. In contrast to *E. coli *RecA, the RecA protein of *D. radiodurans *is able to promote the DNA strand exchange through an inverse pathway, where the double-stranded DNA is bound first and the homologous single-stranded DNA second [43].

The aim of the present study was to clone, sequence and overexpress *D. geothermalis *DSM 11302 and *D. murrayi *DSM 11303 *recA *genes in *E. coli*. A biochemical characterization of recombinant *Dge*RecA and *Dmu*RecA proteins was performed.

## Results

### Cloning, expression and purification of *D. geothermalis *and *D. murrayi *RecA proteins

The primers for amplification of *D. geothermalis *DSM 11302 *recA *gene were designed on the basis of the known *recA *gene sequence of *D. geothermalis *DSM 11300 [GenBank: CP000359]. The obtained PCR product was cloned into a PCR-Blunt vector and sequenced. The nucleotide sequence of *D. geothermalis *DSM 11302 *recA *gene is available from the GenBank database under accession number EF447285.

The predicted *Dge*RecA monomer protein contains 358 amino acid residues. A homology search performed using a version 3 FASTA programme at the EBI (European Bioinformatics Institute) revealed that the amino acid sequence of *D. geothermalis *DSM 11302 RecA shares 100% identity with *D. geothermalis *DSM 11300 RecA protein [GenBank: ABF46432], 87.6% identity and 95.9% similarity with *D. radiodurans *R1 RecA protein [GenBank: AAF11887], 71.1% identity and 91.6% similarity with *Thermus aquaticus *YT-1 RecA protein [GenBank: AAA19796], 71.0% identity and 89.8% similarity with *T. thermophilus *HB8 or *T. thermophilus *HB27 RecA proteins [GenBank: BAD71641 and AAS81808, respectively], 70.6% identity and 89.8% similarity with *Meiothermus ruber *DSM 1279 RecA protein [GenBank: ADD27511], 67.6% identity and 87.2% similarity with *M. silvanus *DSM 9946 RecA protein [GenBank: ADH62770], and 60.9% identity and 84.1% similarity with *E. coli *RecA protein [GenBank: CAA23618].

The *D. murrayi *DSM 11303 *recA *gene sequence was obtained using a two step procedure. In first step, the internal fragment of the *recA *gene was amplified using degenerated primers designed on the basis of an alignment of *recA *gene sequences from bacteria belonging to the *Deinococcus*-*Thermus *group. In the second step, flanking regions were amplified by inverse PCR. The obtained partial sequences of the *D. murrayi recA *gene were then aligned and the primers for amplification of the gene were designed. Afterwards, the PCR product was cloned using a CloneJET™ PCR Cloning Kit and sequenced. The nucleotide sequence of *D. murrayi *DSM 11303 *recA *gene was deposited in the GenBank database under accession number HM004587.

**Figure 1:**
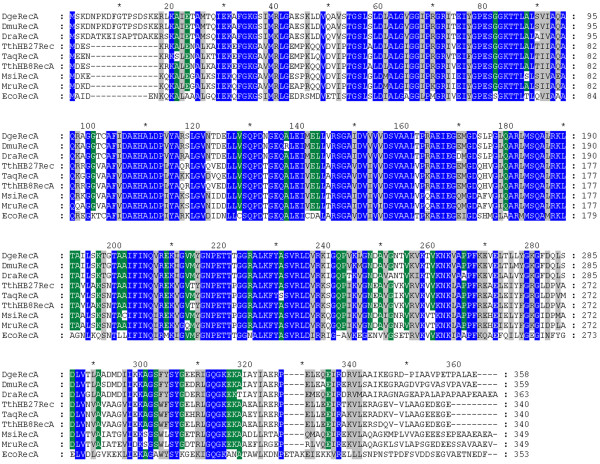
**Multiple sequence alignment of RecA proteins from bacteria belonging to the *Deinococcus*-*Thermus *group and *E. coli *RecA protein**. DgeRecA - *Deinococcus geothermalis *DSM 11302 RecA, DmuRecA - *D. murrayi *DSM 11303 RecA, DraRecA - *D. radiodurans *R1 RecA, TthHB27Rec - *Thermus thermophilus *HB27 RecA, TaqRecA - *T. aquaticus *YT-1 RecA,TthHB8RecA - *T. thermophilus *HB8 RecA, MsiRecA - *Meiothermus silvanus *DSM 9946 RecA, MruRecA - *M. ruber *DSM 1279 RecA, EcoRecA - *E. coli *RecA. Three levels of conserved residues are indicated by blue (100%), green (80%) and grey (60%) backgrounds. The alignment was performed using Clustalx 2.0.11 program.

The *Dmu*RecA protein contains 359 amino acid residues. The deduced amino acid sequence of *D. murrayi *DSM 11303 RecA shows 94.4% identity and 97.8% similarity with *D. geothermalis *DSM 11300 RecA protein, 86.9% identity and 96.9% similarity with *D. radiodurans *R1 RecA protein, 71.4% identity and 90.7% similarity with *Thermus aquaticus *YT-1 RecA protein, 70.7% identity and 89.2% similarity with *T. thermophilus *HB8 RecA protein, 70.3% identity and 89.5% similarity with *Meiothermus ruber *DSM 1279 RecA protein, 69.6% identity and 87.8% similarity with *T. thermophilus *HB27 RecA protein, 67.0% identity and 85.7% similarity with *M. silvanus *DSM 9946 RecA protein, and 62.3% identity and 86.0% similarity with *E. coli *RecA protein. The multiple sequence alignment of RecA proteins from bacteria of the genus *Deinococcus*, *Thermus *and *Meiothermus*, and *E. coli *RecA protein is shown in Figure 1.

**Figure 2:**
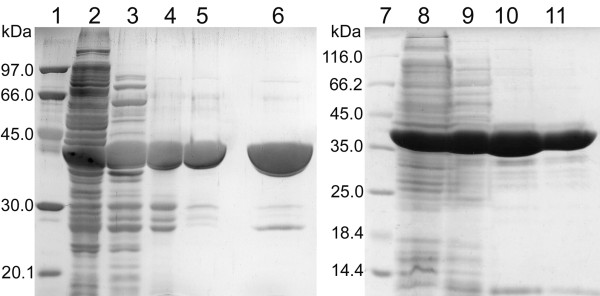
**SDS-PAGE analysis of the fractions obtained by expression and purification of *Dge*RecA and *Dmu*RecA proteins**. Lane 1 - LMW SDS Marker (Amersham Biosciences AB): 97, 66, 45, 30, 20.1 and 14.4 kDa, lane 2 - cell extract of the *E. coli *BLR(DE3) + pET-30Ek/LIC-*Dge*RecA, lane 3 - *Dge*RecA after heat treatment, lane 4 - *Dge*RecA after chromatography on Fractogel EMD DEAE column, lane 5 - *Dge*RecA after chromatography on ResourceQ column, lane 6 - *Dge*RecA after chromatography on MonoQ column, lane 7 - Unstained Protein Molecular Weight Marker (Fermentas): 116, 66.2, 45, 35, 25, 18.4 and 14.4 kDa, lane 8 - cell extract of *E. coli *BLR(DE3) + pET-30Ek/LIC-*Dmu*RecA, lane 9 - *Dmu*RecA after chromatography on Fractogel EMD DEAE column, lane 10 - *Dmu*RecA after chromatography on ResourceQ column, lane 11 - *Dmu*RecA after chromatography on MonoQ column.

In the next step, recombinant plasmids (pET-30Ek/LIC-*Dge*RecA and pET-30Ek/LIC-*Dmu*RecA for biosynthesis of RecA proteins from *D. geothermalis *and *D. murrayi *in *E. coli *T7 expression system) were constructed. After *recA *gene expression in the *E. coli *BLR(DE3) (*recA*^-^) cells, *Dge*RecA protein was purified by heat treatment of lysate at 60°C for 20 min and ion exchange chromatography. In the case of purification of *Dmu*RecA protein heat treatment was omitted due to its low thermostability. The purity of *Dge*RecA and *Dmu*RecA proteins at every step of production and purification was checked by SDS-PAGE after Coomassie Brilliant Blue R staining (Figure 2).

The applied overexpression and purification systems produced about 63 mg of *Dge*RecA and 50 mg of *Dmu*RecA proteins from 1 L of *E. coli *culture.

Analysis of the purified proteins by SDS-PAGE revealed major bands with a molecular mass of about 40 kDa for both *Dge*RecA and *Dmu*RecA proteins (Figure 2, lanes 6 and 11), which agreed with the amino acid sequence calculation; 38.157 kDa and 38.170 kDa, respectively. Summary of the purification process is shown in Table 1.

**Table 1:** Summary of the purification of *Dge*RecA and *Dmu*RecA proteins obtained from 1 L of *E. coli *BLR(DE3) culture.

Protein	Purification step	Total protein (mg)	Recovery of protein (%)
*Dge*RecA	Cell extract	760	100
	Heat treatment	341	45
	Fractogel EMD	121	16
	DEAE column		
	ResourceQ column	75	10
	MonoQ column	63	8

*Dmu*RecA	Cell extract	510	100
	Fractogel EMD	356	70
	DEAE column		
	ResourceQ column	93	18
	MonoQ column	50	10

### Oligomeric states of *Dge*RecA and *Dmu*RecA proteins

**Figure 3:**
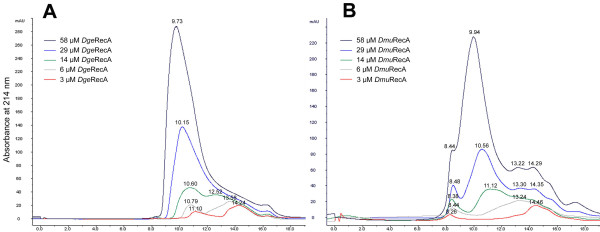
**Effects of *Dge*RecA (A) and *Dmu*RecA (B) proteins concentrations on the elution profiles of gel filtration**. In panel A elution volume of 9.73 ml corresponding to the 14-meric protein; elution volume of 10.15 ml corresponding to the 12-meric protein; elution volumes of 10.60 and 12.52 ml corresponding to 10-meric and tetrameric proteins, respectively; elution volumes of 10.79 and 13.58 ml corresponding to 9-meric and trimeric proteins, respectively; elution volumes of 11.10 and 14.24 ml corresponding to octameric and dimeric proteins, respectively. In panel B elution volumes of 8.44, 8.48, 8.38 and 8.26 ml corresponding to protein agregates; elution volumes of 9.94, 13.22 and 14.29 ml corresponding to 13-meric, trimeric and dimeric proteins, respectively; elution volumes of 10.56, 13.30 and 14.35 ml corresponding to 10-meric, trimeric and dimeric proteins, respectively; elution volume of 11.12 ml corresponding to the octameric protein; elution volume of 13.24 ml corresponding to the trimeric protein; elution volume of 14.46 ml corresponding to the dimeric protein.

Oligomeric states of *Dge*RecA and *Dmu*RecA proteins at a concentration range between 3 and 58 μM in 25 mM potassium phosphate buffer pH 7.5 containing 1 M KCl were analyzed by gel filtration. The oligomeric states of examined proteins depend highly on their level of concentration. At high concentrations in solutions (58 and 29 μM) both RecA proteins exist as a heterogenous population of oligomeric forms ranging in size from dimers (elution volume near 14 ml) to long protein filaments and highly aggregated structures (elution near the void volume of 8.25 ml). Furthermore in such conditions, a greater percentage of *Dge*RecA and *Dmu*RecA proteins exist as big oligomers, although in the case of *Dmu*RecA significant amounts of dimers and trimers (elution volume near 14 and 13 ml, respectively) were also present. At 6 and 3 μM concentrations both proteins eluted at volumes corresponding mainly to the small oligomers and probably monomers in the case of *Dmu*RecA protein (Figure 3).

### ssDNA-binding properties

**Figure 4:**
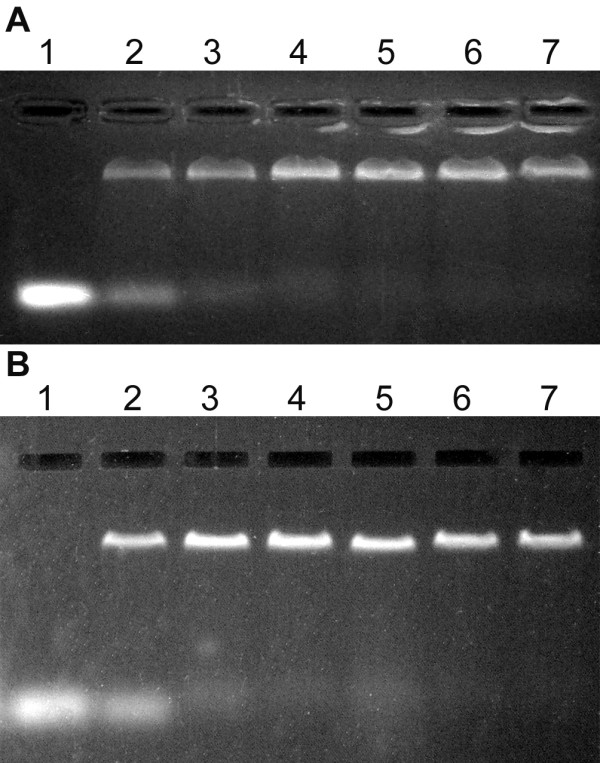
**Binding of *Dge*RecA (A) and *Dmu*RecA (B) to 5'-end fluorescein-labeled (dT)_35_-oligonucleotides in the presence of various Mg^2+ ^ions concentrations at 37°C - gel mobility shift assays**. Lane 1 - oligo(dT)_35_, lane 2 - 0 mM Mg^2+^, lane 3 - 2 mM Mg^2+^, lane 4 - 4 mM Mg^2+^, lane 5 - 6 mM Mg^2+^, lane 6 - 8 mM Mg^2+^, lane 7 - 10 mM Mg^2+^.

**Figure 5:**
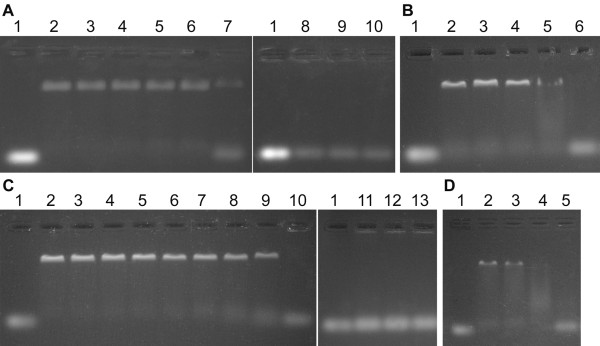
**Binding of *Dge*RecA (A, B) and *Dmu*RecA (C, D) to 5'-end fluorescein-labeled (dT)_35_-oligonucleotides in the presence of 10 mM Mg^2+ ^at various temperatures - gel mobility shift assays**. Panel A: lane 1 - oligo(dT)_35_, lane 2 - 25.0°C, lane - 3 - 31.1°C, lane 4 - 38.2°C, lane 5 - 45.3°C, lane 6 - 51.1°C, lane - 7 - 58.0°C, lane 8 - 65.3°C, lane 9 - 72.2°C, lane 10 - 75.0°C; panel B: lane 1 - oligo(dT)_35_, lane 2 - 55.0°C, lane 3 - 55.9°C, lane 4 - 57.1°C, lane 5 - 58.0°C, lane 6 - 59.4°C; panel C: lane 1 - oligo(dT)_35_, lane 2 - 25.0°C, lane - 3 - 25.9°C, lane 4 - 28.7°C, lane 5 - 33.1°C, lane 6 - 37.9°C, lane - 7 - 42.6°C, lane 8 - 47.4°C, lane 9 - 52.1°C, lane 10 - 56.9°C, lane 11 - 61.2°C, lane 12 - 64.1°C, lane 13 - 65.0°C; panel D: lane 1 - oligo(dT)_35_, lane 2 - 52.0°C, lane 3 - 53.2°C, lane 4 - 54.5°C, lane 5 - 55.6°C.

To determine the ability of *Dge*RecA and *Dmu*RecA proteins to bind ssDNA, we carried out agarose gel mobility assays with 5'-end fluorescein-labelled (dT)_35_-oligonucleotides. The assays were carried out with increasing concentration of Mg^2+ ^ions and at various temperatures between 25 and 75°C. We observed, that ssDNA binding by RecA proteins was stimulated by Mg^2+ ^ions and was the most efficient at 8 and 10 mM Mg^2+ ^(Figure 4). *Dge*RecA was able to bind oligo(dT)_35 _at temperatures between 25 and 58°C while *Dmu*RecA exhibited activity between 25 and 54.5°C, although at the highest temperatures, 58°C for *Dge*RecA and 54.5°C for *Dmu*RecA, the ssDNA binding was very slight (Figure 5).

### dsDNA-binding properties

**Figure 6:**
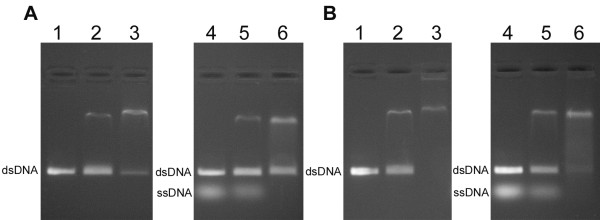
**Binding of *Dge*RecA (A) and *Dmu*RecA (B) to dsDNA in the absence and in the presence of heterologous ssDNA - gel mobility shift assays**. Lane 1 - 600 bp PCR product, lane 2 - dsDNA binding products obtained in the absence of Mg^2+ ^ions, lane 3 - dsDNA binding products obtained in the presence of 10 mM Mg^2+ ^ions, lane 4 - 600 bp PCR product and 5'-end fluorescein-labeled oligo(dT)_35_, lane 5 - DNA binding products obtained in the absence of Mg^2+ ^ions, lane 6 - DNA binding products obtained in the presence of 10 mM Mg^2+ ^ions.

We examined also the ability of RecA proteins from *D. geothermalis *and *D. murrayi *to bind dsDNA in the absence of magnesium ions and in the presence of 10 mM Mg^2+^. Both proteins were able to bind 600 bp PCR products and the dsDNA binding was stimulated by Mg^2+ ^ions as well as in the case of ssDNA binding (Figure 6A and 6B, lanes 2 and 3). Afterwards, to determine what kind of DNA is more readily bound by *Dge*RecA and *Dmu*RecA proteins we carried out the reactions where 5'-end fluorescein-labelled oligo(dT)_35 _and 600 bp PCR product were in the same test tube. When ssDNA and dsDNA were included in the reaction mixture, ssDNA was bound preferentially by both RecA proteins, both in the absence and presence of magnesium ions (Figure 6A and 6B, lanes 5 and 6). We observed also that *Dmu*RecA bound dsDNA more efficient than *Dge*RecA in all conditions tested.

### Thermostability of *Dge*RecA and *Dmu*RecA proteins

Thermostability of RecA proteins was characterized as the longest time period needed for complete loss of visible ssDNA binding activity investigated by gel-mobility shift assay after incubation at given temperature. *Dge*RecA and *Dmu*RecA were incubated at temperatures between 50 and 80°C for 10 s - 180 minutes, in the absence or in the presence of 10 mM Mg^2+^. No significant loss of protein activity was observed after incubation at 50°C for 180 min. *Dge*RecA was also stable for 180 min at 57°C, while *Dmu*RecA lost activity within 15 min at this temperature. Both proteins incubated at higher temperatures displayed lower thermostability and at 80°C were completely inactivated in 10 s. The RecA protein from *D. geothermalis *was more thermostable than RecA from *D. murrayi *(Table 2). Moreover magnesium ions have no effect on the thermal stability neither *Dge*RecA nor *Dmu*RecA.

**Table 2:** Thermostability of *Dge*RecA and *Dmu*RecA proteins.

Temperature (°C)	Maximum incubation time (min)	
	*Dge*RecA	*Dmu*RecA
50	180	180
57	180	15
62	20	8
71	1	0.67
75	0.50	0.33
80	0^a^	0^a^

^a ^Complete lost of activity after shorter than 10 s incubation time

### ATP and dATP hydrolysis

ATP and dATP hydrolysis catalyzed by RecA proteins from *D. geothermalis *and *D. murrayi *was monitored with a coupled spectrophotometric assay. We studied the DNA and temperature dependence of *Dge*RecA and *Dmu*RecA activity. We also measured the ATPase activity of both proteins at various concentrations of ATP and dATP to determine their kinetic parameters.

We found no detectable ATPase and dATPase activities of *Dge*RecA and *Dmu*RecA in the absence of DNA and in the presence of dsDNA only. Both proteins however were able to hydrolyze ATP and dATP in the presence of ssDNA and in the DNA strand exchange conditions, although the rates of ssDNA-dependent ATP or dATP hydrolysis were higher. Furthermore, both proteins hydrolyzed dATP more rapidly than ATP either in the presence of ssDNA or ssDNA and dsDNA in the same reaction mixture (Tables 3 and 4). The rates of ssDNA-dependent ATP and dATP hydrolysis increased with temperature for both proteins and peaked at 42°C and 45°C for *Dmu*RecA and *Dge*RecA, respectively (Table 5). Kinetic parameters for ATPase and dATPase activity of *Dge*RecA and *Dmu*RecA proteins are displayed in Tables 6 and 7.

**Table 3:** Effect of DNA on the rate of ATP and dATP hydrolysis catalyzed by *Dge*RecA.

Substrate	Rate of hydrolysis (μM/min)			
	absence of DNA	dsDNA	ssDNA	ssDNA and dsDNA^a^
ATP	not detected	not detected	26.8 ± 0.40	20.3 ± 0.36
dATP	not detected	not detected	31.6 ± 0.63	23.7 ± 0.43

Reactions conditions: 37°C, 2.27 mM ATP or dATP.

^a ^DNA strand exchange conditions

**Table 4:** Effect of DNA on the rate of ATP and dATP hydrolysis catalyzed by *Dmu*RecA.

Substrate	Rate of hydrolysis (μM/min)			
	absence of DNA	dsDNA	ssDNA	ssDNA and dsDNA^a^
ATP	not detected	not detected	34.4 ± 0.58	21.2 ± 0.44
dATP	not detected	not detected	47.0 ± 0.71	24.6 ± 0.49

Reactions conditions: 37°C, 2.27 mM ATP or dATP.

^a ^DNA strand exchange conditions

**Table 5:** Effect of temperature on the rate of ssDNA-dependent ATP and dATP hydrolysis catalyzed by *Dge*RecA and *Dmu*RecA.

Protein	Substrate	Rate of hydrolysis (μM/min)				
		32°C	37°C	42°C	45°C	50°C
*Dge*RecA	ATP	19.8 ± 0.26	26.8 ± 0.40	31.2 ± 0.56	34.1 ± 0.78	26.6 ± 0.43
	dATP	10.3 ± 0.18	31.6 ± 0.63	53.7 ± 1.18	65.7 ± 1.71	56.5 ± 1.30

*Dmu*RecA	ATP	15.6 ± 0.23	34.4 ± 0.58	55.2 ± 1.16	47.6 ± 1.20	34.5 ± 0.62
	dATP	24.2 ± 0.34	47.0 ± 0.71	70.3 ± 1.76	59.9 ± 1.38	43.9 ± 0.88

Reaction conditions: 2.27 mM ATP or dATP.

**Table 6:** Kinetic parameters for ATP and dATP hydrolysis catalyzed by *Dge*RecA.

Substrate	DNA	T (°C)	K_M _(mM)	k_cat _(s^-1^)	k_cat_/K_M _(s^-1 ^M^-1^)	n^a^
ATP	ssDNA	37	0.56 ± 0.018	0.15 ± 0.004	2.7 ± 0.12 × 10^2^	2.9
	ssDNA and dsDNA	37	0.13 ± 0.006	0.13 ± 0.006	1.0 ± 0.04 × 10^3^	1.4

dATP	ssDNA	37	0.62 ± 0.023	0.21 ± 0.007	3.3 ± 0.10 × 10^2^	1.2
	ssDNA and dsDNA	37	0.36 ± 0.016	0.15 ± 0.007	4.2 ± 0.16 × 10^2^	1.0

^a ^Hill coefficient

**Table 7:** Kinetic parameters for ATP and dATP hydrolysis catalyzed by *Dmu*RecA.

Substrate	DNA	T (°C)	K_M _(mM)	k_cat _(s^-1^)	k_cat_/K_M _(s^-1 ^M^-1^)	n^a^
ATP	ssDNA	37	0.31 ± 0.009	0.13 ± 0.004	4.3 ± 0.18 × 10^2^	1.1
	ssDNA and dsDNA	37	0.28 ± 0.009	0.067 ± 0.0021	2.4 ± 0.08 × 10^2^	1.5

dATP	ssDNA	37	0.28 ± 0.007	0.14 ± 0.004	5.0 ± 0.25 × 10^2^	1.0
	ssDNA and dsDNA	37	0.22 ± 0.007	0.073 ± 0.0023	3.4 ± 0.05 × 10^2^	1.3

^a ^Hill coefficient

### DNA strand exchange reactions

**Figure 7:**
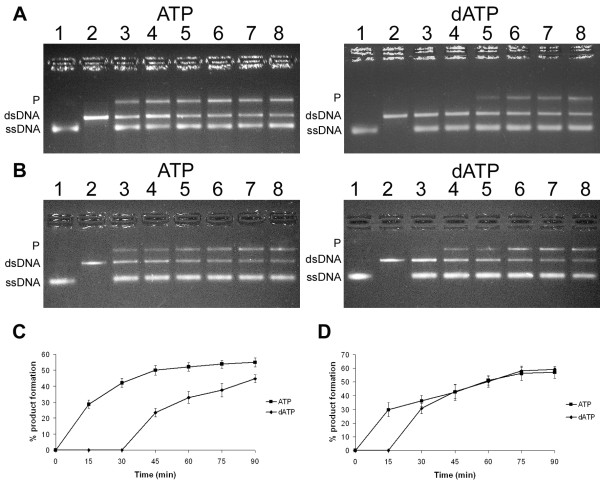
**DNA strand exchange promoted by *Dge*RecA (A, C) and *Dmu*RecA (B, D) proteins**. Panels A and B: lane 1 - M13mp18 ssDNA, lane 2 - M13mp18 dsDNA digested with *Sma*I, lane 3 - reaction products formed after 15 min, lane 4 - reaction products formed after 30 min, lane 5 - reaction products formed after 45 min, lane 6 - reaction products formed after 60 min, lane 7 - reaction products formed after 75 min, lane 8 - reaction products formed after 90 min. P - nicked circular dsDNA. Panels C and D: plots of the quantified product formation for the reactions in panels A and B.

The ability of *Dge*RecA and *Dmu*RecA proteins to promote DNA strand exchange reaction was investigated in the presence of ATP or dATP, ATP-regenerating system and *Dge*SSB protein (obtained according to a procedure by Filipkowski et al. [44]), at 42°C for *Dmu*RecA and 45°C for *Dge*RecA. At first, filaments composed of ssDNA and RecA protein were formed. Afterwards, linear complementary dsDNA was added to start DNA strand exchange. After incubation and at various time periods, reactions were halted by protein degradation. As shown in Figure 7, *Dge*RecA and *Dmu*RecA promoted homologous DNA strand exchange either in the presence of ATP or dATP as evidenced by forming nicked circular dsDNA products, although the products appeared earlier when reactions were performed with ATP. Moreover DNA strand exchange occurred faster when *Dmu*RecA was used. We also performed DNA strand exchange reactions via an inverse pathway, where RecA proteins were preincubated with the linear dsDNA and circular ssDNA was then added to initiate reactions, however we do not observe nicked circular dsDNA products even after 90 min of incubation.

## Discussion

In this article, the cloning, purification and initial characterization of RecA proteins from slightly thermophilic and extremely radioresistant bacteria *D. geothermalis *and *D. murrayi *are described. The *Dge*RecA and *Dmu*RecA exhibit many properties common to this class of proteins. In the absence of DNA both proteins self-assemble into a variety of oligomers, their size strongly depending on the concentration of protein in the solution, a characteristic of RecA proteins. Although oligomeric populations of *T. aquaticus *and *T. thermophilus *RecA proteins contain a large percentage of hexamers [45-48], it was impossible in our study to determine the preferred oligomeric state of *Dge*RecA and *Dmu*RecA.

RecA proteins of *D. geothermalis *and *D. murrayi *bind both ssDNA and dsDNA like other RecA proteins. However, it was demonstrated that RecA protein of *D. radiodurans *binds preferentially to double-stranded DNA even when ssDNA is present in the reaction mixture [49,50]. In contrast to *Dra*RecA, *Dge*RecA and *Dmu*RecA bind more readily to single-stranded DNA when both ssDNA and dsDNA are in the same reaction mixture.

RecA proteins of *D. geothermalis *and *D. murrayi *are DNA-dependent ATPases. In the absence of DNA, ATP or dATP, hydrolysis was not detected. A similar result was demonstrated for *D. radiodurans *RecA [50]. In contrast, ATP hydrolysis in the absence of exogenous DNA was detected for *E. coli *RecA protein, although the rate was significantly reduced [33]. Unlike *Dra*RecA protein [49,50], the *Dge*RecA and *Dmu*RecA proteins were not able to perform ATP or dATP hydrolysis in the presence of dsDNA at pH 7.5. The same results were shown for *T. thermophilus *and *E. coli *RecA proteins [33,51], although *Eco*RecA protein hydrolyses ATP in the presence of dsDNA at lower pH (optimum near pH 6) [33]. The ATP and dATP hydrolysis catalyzed by RecA proteins of *D. geothermalis *and *D. murrayi *was stimulated by single-stranded DNA, and dATP was hydrolyzed faster than ATP. These results are consistent with the results obtained for RecA proteins of *E. coli *and *D. radiodurans *[33,49].

Both *Dge*RecA and *Dmu*RecA as well as *Eco*RecA and *Dra*RecA were able to promote DNA strand exchange using ATP or dATP as a cofactor [41,49]. However, in the presence of dATP nicked circular heteroduplex products formed more slowly than in the presence of ATP. Similar results were also obtained for RecA protein of *D. radiodurans *[49].

The major difference between *E. coli *and *D. radiodurans *RecA proteins is the ability to promote DNA strand exchange using inverse pathways. The *Eco*RecA initiates DNA strand exchange with a filament bound to the ssDNA, while the *Dra*RecA binds dsDNA first and homologous ssDNA second [43]. In our study, we did not obtained the product of DNA strand exchange reactions initiated with *Dge*RecA-dsDNA or *Dmu*RecA-dsDNA filaments, although Sghaier et al. [52] demonstrated that RecA protein of *D. geothermalis *is able to promote DNA exchange reactions through normal and inverse pathways. The difference in results obtained by Sghaier et al. and us may be due to differences in the conditions of the DNA strand exchange reactions, especially the differences in the length of dsDNA fragments used. In our study, we performed DNA strand exchange reactions between full length linear M13mp18 dsDNA (7249 bp) and circular M13mp18 ssDNA, whereas Sghaier et al. used a short fragment of ΦX174 dsDNA obtained by digestion with *Hin*cII endonuclease (the longest DNA fragment obtained by *Hin*cII digestion of ΦX174 RFI and its size was 1057 bp) and circular ΦX174 ssDNA (5386 nt).

RecA proteins of *D. geothermalis *and *D. murrayi *are active at elevated temperatures similarly to RecA of *T. thermophilus*. The optimum temperature for *Tth*RecA ATPase activity is 65°C [51], and for *Dge*RecA and *Dmu*RecA proteins is 45 and 42°C, respectively. However, they are able to bind ssDNA at temperatures reaching 54.5°C for *Dmu*RecA and 58°C for *Dge*RecA. Moreover, the thermostability assay revealed that the *Dge*RecA and *Dmu*RecA are less thermostable than *Tth*RecA which remains still active after a 10-s incubation at 85°C (data not shown), whereas *Dge*RecA and *Dmu*RecA are completely inactivated in 10 s at 80°C.

## Conclusions

The properties of RecA proteins of slightly thermophilic and extremely radioresistant bacteria *D. geothermalis *and *D. murrayi *obtained in this study demonstrate that they are close functional homologues of the other RecA proteins. Although it has previously been shown that the RecA protein of the extremely radioresistant bacterium *D. radiodurans *exhibits some unusual properties, in our study we did not observe such properties in the case of *Dge*RecA and *Dmu*RecA. This suggests the uniqueness of *Dra*RecA even among RecA proteins from microorganisms of the *Deinococcus *genus. On the other hand, it has been also demonstrated that the dsDNA-binding preferences displayed by *Dra*RecA are not necessary for double-stranded breaks repair in γ-irradiated *D. radiodurans*. The rapid reconstruction of an intact genome occurring through an extended synthesis-dependent strand annealing process (ESDSA) followed by a DNA recombination requires a 5'-3' single-stranded DNA exonuclease RecJ, a RecQ helicase and a RecF, RecO and RecR proteins to act together to promote loading of RecA onto single-stranded DNA [53].

## Methods

### Bacterial strains and growth conditions

*D. geothermalis *DSM 11302 and *D. murrayi *DSM 11303 were purchased from Deutsche Sammlung von Mikroorganismen und Zellkulturen (Braunschweig, Germany). They were grown in 50 ml modified Luria-Bertani medium pH 7.2 containing 1 g peptone K, 1 g yeast extract and 7.4 g sea salt in 1000 ml distilled water. *D. geothermalis *was grown for 30 h at 50°C and *D. murrayi *was grown for 30 h at 47°C. Afterwards genomic DNA was isolated using Genomic Mini AX Bacteria (A&A Biotechnology, Poland).

### Cloning of the *D. geothermalis recA *gene

The *recA *gene was amplified by PCR using genomic DNA from *D. geothermalis *DSM 11302 as the template. The primers used were: DGRAFNde 5' CGACAT**ATG**AGCAAGGAACAACCCCAAGGA 3', containing recognition site for *Nde*I endonuclease (underlined) and DGRARHnd 5' ACAAAGC**TTA**CTCTGCCAAGGCGGGC 3', containing recognition site for *Hin*dIII endonuclease (underlined). The start and stop codons are bolded. The reaction mixture consisted of 0.13 μg of *D. geothermalis *DNA, 0.2 μM of each primer, 200 μM of each dNTP, 2 mM MgSO_4 _and 1 U of *Delta3 *DNA polymerase (DNA-Gdańsk, Poland) in 1 × PCR buffer (20 mM Tris-HCl pH 8.8, 10 mM KCl, 10 mM (NH_4_)_2_SO_4_, 0.1% Triton X-100). PCR reaction was performed using the following conditions: 95°C - 2 min, (95°C - 1 min, 61°C - 1 min, 72°C - 1 min; 30 cycles), 72°C - 5 min. The PCR product was cloned into the pCR-Blunt vector (Invitrogen, Carlsbad, California, USA) and sequenced. The obtained pCR-Blunt-NdeI-*Dge*RecA-HindIII plasmid was then digested with *Nde*I and *Hin*dIII endonucleases, and the DNA fragment containing the *recA *gene was cloned into pET-30 Ek/LIC expression vector (Novagen, Beeston, Nottingham, England) digested with the same restriction enzymes. The resulting recombinant plasmid pET-30Ek/LIC-*Dge*RecA was used for the production of *D. geothermalis *RecA protein in *E. coli*.

### Isolation and cloning of the *D. murrayi recA *gene

In order to obtain an internal part of the *recA *gene from *D. murrayi *DSM 11303, sequences encoding RecA proteins of *Deinococcus geothermalis *DSM 1300, *D. geothemalis *DSM 11302, *Deinococcus radiodurans *R1, *Thermus aquaticus *YT-1, *Thermus thermophilus *HB8 and *T. thermophilus *HB27 obtained from the GenBank database were aligned using the ClustalX program, version 1.8. Based on the alignment, degenerated primers InvRecA1 5' GAGTCSGGSGGCAAGACCAC 3' and InvRecA2 5' TCCTTSCCCTGGCCSAKGC 3' were designed and synthesized. The PCR reaction was performed in the mixture containing: 0.2 μM of each primer, 0.2 μg of *D. murrayi *DSM 11303 genomic DNA, 200 μM of each dNTP, 3 mM MgCl_2 _and 1 U of DNA polymerase *Hypernova *(DNA-Gdańsk, Poland) in 1 × buffer *Hypernova *(10 mM Tris-HCl pH 8.8, 50 mM KCl, 0.15% Triton X-100). The reaction mixture was incubated for 2 min at 95°C, followed by 30 cycles at 95°C for 1 min, 59°C for 1 min, 72°C for 1 min, and a final incubation for 5 min at 72°C. The obtained PCR product was then purified from an agarose gel band using Gel-Out kit (A&A Biotechnology, Poland), cloned into pJET1.2/blunt vector CloneJET™ PCR Cloning Kit (Fermentas, Vilnius, Lithuania) and sequenced. Afterwards, the inverse PCR was performed for obtaining of flanking regions. The *D. murrayi *genomic DNA was first digested with *Dra*I restriction endonuclease and then restriction fragments were ligated upon itself to form circles. In the second step, the PCR amplification was performed using ligation products as a template and two primers: recAInvFor 5' CCGCAAGATTGGGCAGCCCGTCAAGA 3' and recAInvRev 5' ACCCGACCGCACGAGCAGCTCCATG 3', designed on the basis of previously obtained partial sequence of *D. murrayi recA *gene. The reaction mixture contained also 200 μM of each dNTP, 3 mM MgCl_2 _and 1 U of DNA polymerase *Hypernova *(DNA-Gdańsk, Poland) in 1 × buffer *Hypernova*. DNA amplification was performed using the following conditions: 95°C - 3 min, (95°C - 1.5 min, 66°C - 1 min, 72°C - 5 min) 30 cycles and 72°C - 15 min after the final cycle. The PCR product was purified from an agarose gel band, cloned into pJET1.2/blunt vector and sequenced. Afterwards, fragments of *D. murrayi *genomic DNA sequence were alignment and the full sequence of *recA *gene was obtained.

The *D. murrayi *DSM 11303 *recA *gene was then amplified using the forward primer FDMRecANdeI 5' ATTACAT**ATG**AGCAAGGACAACCCCAAGGACTTC 3', and the reverse primer RDMRecAXhoI 5' TATTCTCGAG**TTA**CTCCGCGACAGCGGGCAC 3', containing *Nde*I and *Xho*I recognition sites, respectively (underlined). The start and stop codons are bolded. The PCR reaction mixture contained: 0.2 μM of each primer, 0.2 μg of *D. murrayi *genomic DNA, 200 μM of each dNTP, 3 mM MgCl_2 _and 1 U of DNA polymerase *Hypernova *(DNA-Gdańsk, Poland) in 1 × *Hypernova *buffer. The reaction mixture was incubated for 3 min at 96°C, followed by 5 cycles at 95°C for 1 min, 58°C for 1 min, 72°C for 1 min and 25 cycles at 95°C for 1 min, 63°C for 1 min, 72°C for 1 min, and a final incubation for 5 min at 72°C. Afterwards, PCR product was purified from an agarose gel band, cloned into pJET1.2/blunt vector and sequenced. The obtained pJET-NdeI-*Dmu*RecA-XhoI plasmid was then digested with *Nde*I and *Xho*I endonucleases, and the DNA fragment containing the *recA *gene was cloned into pET-30 Ek/LIC expression vector (Novagen) digested with the same restriction enzymes. The resulting recombinant plasmid pET-30Ek/LIC-*Dmu*RecA was used for the production of *D. murrayi *RecA protein in *E. coli*.

### Production and purification of recombinant *Dge*RecA and *Dmu*RecA proteins

Overproduction of *Dge*RecA and *Dmu*RecA proteins was performed in the *E. coli *BLR(DE3) cells (Novagen) carrying the pET-30Ek/LIC-*Dge*RecA or pET-30Ek/LIC*Dmu*RecA plasmids. Cells were grown at 37°C in 750 ml LB medium (1% peptone K, 0.5% yeast extract, 1% NaCl) containing 20 μg/ml of kanamycin. At OD_600 _0.5 IPTG was added to the final concentration of 1 mM and cultivation was continued for 4 hours. Subsequently, cultures were centrifuged (4612 × *g*, 10 min, 4°C) and pellets were resuspended in 75 ml of buffer A_1 _(20 mM potassium phosphate buffer pH 6.0, 50 mM KCl, 1 mM EDTA, 1 mM DTT) in the case of *Dge*RecA protein production or buffer A_2 _(20 mM potassium phosphate buffer pH 6.5, 50 mM KCl, 1 mM EDTA, 1 mM DTT ) for the *Dmu*RecA. Samples were sonicated seven times for 30 s at 0°C and centrifuged (4612 × *g*, 10 min, 4°C). The supernatant containing *Dge*RecA protein was heat-treated at 60°C for 20 min, cooled on ice and centrifuged again (18000 × *g*, 30 min, 4°C).

*Dge*RecA and *Dmu*RecA proteins were then purified using Fractogel EMD DEAE column (Merck, Darmstadt, Germany) equilibrated with buffer A_1 _or A_2_, respectively. RecA proteins were eluted with linear gradient of 0.05-1.5 M KCl in the appropriate buffer. Fractions containing *D*. geothermalis or *D. murrayi *RecA were pooled, dialyzed against buffer A_1 _or A_2 _and loaded onto ResourceQ column (Amersham Biosciences AB, Uppsala, Sweden). Elution was performed by linear gradient of 0.05-0.425 M KCl in buffer A_1 _or A_2_. Fractions containing RecA proteins were dialyzed against buffer A_1 _or A_2 _once again and purified using MonoQ column (Amersham Biosciences AB). *Dge*RecA and *Dmu*RecA were eluted with linear gradient of 0.05-0.35 M KCl in appropriate buffer. Collected fractions containing purified RecA proteins were then checked for nucleases contaminations by incubating them with M13mp18 RFI DNA (24 μM of nucleotides), linear dsDNA M13mp18 (24 μM of nucleotides) and circular ssDNA M13mp18 (12 μM of nucleotides) in the 20 mM potassium phosphate buffer pH 7.5 containing 10 mM MgCl_2_. The concentration of RecA proteins was 10 μM. Reaction mixtures were incubated at 37°C for 2 h and then EDTA, SDS and Proteinase K were added to the final concentrations of 10 mM, 1% and 2 mg/ml, respectively. Samples were incubated at room temperature for 30 min and separated by electrophoresis in 1% agarose gels with ethidium bromide. DNAs were also incubated with Proteinase K only, as a control sample. We did not observe any exo- or endonuclease activities neither in RecA proteins preparations nor in Proteinase K. Afterwards the RecA proteins were dialyzed against 100 mM (NH_4_)HCO_3 _and lyophilized for long-time storage.

The purity of proteins was investigated by SDS-PAGE. The concentration of RecA proteins at every step of production and purification was measured by Bradford method [54]. Protein concentration in the samples for biochemical tests was determined by A_280 nm _using an extinction coefficient of 11,920 M^-1 ^cm^-1^, which was calculated from the amino acid sequences of *Dge*RecA and *Dmu*RecA proteins.

### Estimation of the native molecular mass

The molecular mass of *Dge*RecA and *Dmu*RecA proteins were estimated using analytical gel filtration chromatography. Samples containing various concentrations of purified proteins were loaded onto Superdex 200 HR 10/30 column (Amersham Biosciences AB) equilibrated with 25 mM potassium phosphate buffer pH 7.5 containing 1.0 M KCl and eluted with the same buffer at a flow rate of 0.5 ml/min. The elution profiles were monitored by recording the absorbance at 214 nm. The molecular weights of *Dge*RecA and *Dmu*RecA oligomers were determined by comparison with those of standard proteins: thyroglobulin (669 kDa), apoferritin (443 kDa), β-amylase (200 kDa), alcohol dehydrogenase (150 kDa), bovine serum albumin (66 kDa) and carbon anhydrase (29 kDa).

### ssDNA binding activity

The ability of *Dge*RecA and *Dmu*RecA proteins to bind ssDNA was investigated by electrophoretic mobility shift of fluorescein 5'-end-labelled oligo(dT)_35 _nucleotides. 25 μl reaction mixtures containing 5.6 μM (nucleotides) oligo(dT)_35_, 110 μM ATP-γ-S, 300 μM ATP, from 0 to 10 mM MgCl_2 _and 9 μM RecA protein in 20 mM potassium phosphate buffer pH 7.5 were incubated at 25-75°C for 30 min in the dark. Afterwards samples were separated by electrophoresis in 0.8% agarose gels. Results were visualized using UV transilluminator.

### dsDNA binding activity

The ability of *Dge*RecA and *Dmu*RecA proteins to bind dsDNA was investigated by electrophoretic mobility shift of 600 bp PCR product (λ DNA amplified using 5' GGACAAGGTCGCTGAAGCCTTCG 3' and 5' TCCTGACGGGCGGTATATTTCTCC 3' primers). 25 μl reaction mixtures containing 30 μM (nucleotides) dsDNA or 30 μM dsDNA and 14 μM ssDNA (fluorescein 5'-end-labelled oligo(dT)_35_), 180 μM ATP-γ-S, 500 μM ATP, 0 or 10 mM MgCl_2 _and 12 μM RecA protein in 20 mM potassium phosphate buffer pH 7.5 were incubated at 37°C for 30 min. Afterwards samples were separated by electrophoresis in 0.8% agarose gels containing ethidium bromide. Results were visualized using UV transilluminator.

### Thermostability of the RecA proteins

The thermostability of *Dge*RecA and *Dmu*RecA was determined by ssDNA binding activity after incubation of proteins at various temperatures for a set of time periods. The samples containing 11.8 μM RecA protein and 0 or 10 mM MgCl_2 _in 20 mM potassium phosphate buffer pH 7.5 were incubated at 50-80°C for 10 s to 180 min, cooled on ice and centrifuged (13500 × *g*, 10 min). Aliquots (19 μl) were then taken and fluorescein 5'-end-labelled oligo(dT)_35 _nucleotides, ATP, ATP-γ-S and MgCl_2 _were added to the concentrations of 5.6 μM, 300 μM, 110 μM and 10 mM in the final volume of 25 μl, respectively. Subsequently reaction mixtures were incubated at 37°C for 30 min in the dark and separated by electrophoresis in 0.8% agarose gels. Results were visualized using UV transilluminator.

### ATPase assay

Hydrolysis of ATP (or dATP) by *Dge*RecA and *Dmu*RecA was monitored by an enzyme coupled method. Regeneration of ATP from ADP and phosphoenolpyruvate catalyzed by pyruvate kinase was coupled with the conversion of NADH to NAD^+ ^catalyzed by lactate dehydrogenase, which was monitored by a decrease in absorbance at 355 nm. The absorbance was measured using PerkinElmer multilabel plate reader Victor^3 ^V (Centre of Excellence ChemBioFarm, Gdańsk University of Technology, Gdańsk, Poland).

The reaction mixtures consisted of: 3.03 μM RecA protein, 11.56 mM MgCl_2_, 1.25 U of lactate dehydrogenase from rabbit muscle, 0.75 U of pyruvate kinase from rabbit muscle, 3.3 mM NADH, 2.27 mM PEP, 0.38-2.27 mM ATP (or dATP) and 20 mM Tris-HCl buffer pH 7.5 to the end volume of 75 μl. Reactions were carried out in the absence of DNA and in the presence of 12.16 μM (nucleotides) ssDNA of SygB1a oligonucleotide 5' CGTTCGAAACGATGAGGAAGACTTATTGGCTGATGGCGCTTTTCGCGGTGCTCATGTTCCCTGAAAAGTTCCTTTGGGGT 3' (80 nt) and/or 1,312 μM (nucleotides) of *Xba*I-linearized dsDNA of pBADTOPOβAQa plasmid (5727 bp) containing complementary sequence to the SygB1a oligonucleotide. ATP (or dATP) hydrolysis reactions were performed at 32, 37, 42, 45 and 50°C.

### DNA strand exchange reactions

RecA-dependent DNA strand exchange reactions were carried out between circular ssDNA of M13mp18 and linear dsDNA of M13mp18 obtained by *Sma*I digestion. All reactions were carried out in solutions containing 20 mM Tris-HCl buffer pH 7.5, 5 μM RecA protein, 9 μM (nucleotides) ssDNA, 18 μM (nucleotides) dsDNA, 0.3 μM *Dge*SSB protein [44], 1.2 mM DTT, 10 mM MgCl_2_, 3 mM ATP and an ATP-regenerating system (0.5 U pyruvate kinase from rabbit muscle, 3 mM phosphoenolpyruvate) in 25 μl. A preincubation of ssDNA with *Dge*RecA at 45°C or with *Dmu*RecA at 42°C for 5 min was followed by addition of ATP or dATP and SSB. After an additional 5 min incubation, linear dsDNA was added to start the strand exchange reactions. The reactions were carried out at 45°C when *Dge*RecA was used or at 42°C in the case of *Dmu*RecA for 15, 30, 45, 60, 75 and 90 min and stopped by addition of EDTA, SDS and Proteinase K to the final concentrations of 10 mM, 1% and 2 mg/ml, respectively. Samples were incubated at room temperature for 30-90 min and then separated by electrophoresis in 0.8% agarose gels. Results were visualized and photographed with UV light using VersaDoc™ Imaging System (Bio-Rad Laboratories, Hercules, California, USA) after staining of gels with ethidium bromide. The DNA bands were quantified with Quantity One software, version 4.3.1 (Bio-Rad Laboratories). The band corresponding to nicked circular dsDNA product was quantified as the fraction of the total DNA in a given gel lane, excluding the band corresponding to the ssDNA. In the case of inverse DNA strand exchange reactions, RecA proteins were preincubated with the linear dsDNA and ATP or dATP for 15-60 min. The ssDNA was then added to start the reactions, and the SSB was added 5 min later.

## Abbreviations

DSB: DNA double-strand break; *Dge*RecA: *Deinococcus geothermalis *RecA protein; *Dmu*RecA: *Deinococcus murrayi *RecA protein; *Dra*RecA: *Deinococcus radiodurans *RecA protein; *Tth*RecA: *Thermus thermophilus *RecA protein; *Eco*RecA: *Escherichia coli *RecA protein; *Dge*SSB: *Deinococcus geothermalis *single-stranded DNA-binding protein; ATP-γ-S: Adenosine 5'-[γ-thio]triphosphate; IPTG: Isopropyl β-D-thiogalactopyranoside.

## Authors' contributions

MW participated in the design of the study, partially characterized of RecA proteins and drafted the manuscript; PH obtained and partially characterized of RecA proteins; BK partially characterized of RecA proteins; JK corrected and edited the final version of manuscript. All authors read and approved the final manuscript.
